# Epidermal growth factor receptor (EGFR) expression in the serum of patients with triple-negative breast carcinoma: prognostic value of this biomarker

**DOI:** 10.3332/ecancer.2022.1431

**Published:** 2022-07-20

**Authors:** Rogério Agenor de Araújo, Felipe Andrés Cordero da Luz, Eduarda da Costa Marinho, Camila Piqui Nascimento, Lara de Andrade Marques, Patrícia Ferreira Ribeiro Delfino, Rafael Mathias Antonioli, Breno Jeha Araújo, Ana Cristina Araújo Lemos da Silva, Maria Luiza Gonçalves dos Reis Monteiro, Morun Bernardino Neto, Marcelo José Barbosa Silva

**Affiliations:** 1 Federal University of Uberlândia, Avenida Pará, Bloco 2U, 1720, Campus Umuarama, Uberlândia, MG, CEP 38400-902, Brazil; 2 Cancer Research and Prevention Nucleus, Grupo Luta Pela Vida, Cancer Hospital in Uberlândia, Uberlândia, MG, CEP 38405-302, Brazil; 3 São Paulo State Cancer Institute of the Medical School of the University of São Paulo, São Paulo, SP, CEP 38405-302, Brazil; 4 Department of Basic and Environmental Sciences, University of São Paulo, Lorena, SP, CEP 12602-810, Brazil; 5 Laboratory of Tumor Biomarkers and Osteoimmunology, Institute of Biomedical Sciences, Federal University of Uberlândia, Uberlândia, MG, CEP 38405-320, Brazil; This study was published as an online abstract at the 2021 American Society of Clinical Oncology (ASCO) Annual Meeting.; a https://orcid.org/0000-0003-4653-6786; b https://orcid.org/0000-0002-9381-4913; c https://orcid.org/0000-0002-1307-9104; d https://orcid.org/0000-0002-0955-8559; e https://orcid.org/0000-0002-2734-8352; f https://orcid.org/0000-0002-2196-9318; g https://orcid.org/0000-0003-3886-1562; h https://orcid.org/0000-0003-4892-9911; i https://orcid.org/0000-0002-8220-938X; j https://orcid.org/0000-0002-7961-6996; k https://orcid.org/0000-0003-4292-7800; l https://orcid.org/0000-0002-5807-4286

**Keywords:** breast cancer, prognosis, sEGFR, tEGFR

## Abstract

**Background:**

Epidermal growth factor receptor (EGFR) overexpression has been considered a poor prognostic factor in breast cancer.

**Methodology:**

A prospective study of 206 women with breast cancer analysed by stages (I, II, III and IV) and by immunohistochemical subtype (Luminal A, Luminal B, HER2+ and triple-negative (TN)); 89 healthy controls with normal recent mammography were included. The EGFR measured in the serum (sEGFR) was detected by the Enzyme-Linked Immunosorbent Assay (ELISA) method (R&D Systems kit DY231) collected by blood before any treatment in patients. Kaplan–Meier method and Cox regression were carried out to obtain the prognostic value, considering significance if p < 0.05.

**Results:**

With a median follow-up of 36.6 months, 47 deaths occurred. Multivariable Cox regression showed difference of overall survival (OS) associated with sEGFR levels (sEGFR ≤ or > 47.8 ng/mL) in patients with TN cancers, but not of Luminal A, Luminal B or HER2+ subtypes; adjusted by stage, the death risk increased by approximately 415% [hazard ratio (HR): 5.149 (1.900–13.955), p = 0.001] for patients with sEGFR > 47.8 ng/mL compared to patients with a lower sEGFR value. There was no significant correlation of sEGFR with staging, histological tumour grade (G1/G2/G3), Ki67 (< or ≥14%) or body mass index.

**Conclusions:**

Increased sEGFR expression in patients with TN tumours is a significant predictor of lower OS and its quantification is inexpensive and straightforward.

## Introduction

Breast cancer has a high mortality rate [1], but the prognosis can be improved if early diagnosis is made possible by mammography in older women [2], or by magnetic resonance imaging [3] in younger women [4]. After histological confirmation, TNM staging is performed and tumour subtypes are defined by immunohistochemical (IH), with hormone therapy for patients with luminal tumours [5], and monoclonal antibodies in those with Human Epidermal Receptor type 2 (HER2) overexpression [6], in addition to chemotherapy. However, for patients with triple-negative (TN) tumours, there are far fewer therapeutic options [5]. There are risks of recurrence at all subtypes, even in the early stages [7], requiring continuous treatment improvements [8].

Epidermal growth factor receptor (EGFR) is a glycoprotein, expressed as a transmembrane tyrosine kinase receptor, is studied as a biomarker in TN breast tumours, which can be expressed in 200,000–400,000 receptors in malignant cells. The EGFR has a half-life of 10 hours, undergoing intense recycling due to endocytosis, mainly by clathrin [9] and can be internalised in the cell nucleus. EGFR acts on gene transcription, increasing proliferation, reducing DNA damage by chemotherapy, and thus favouring the resistance to treatment [10]. EGFR can be measured by IH, Fluorescence In Situ Hybridization (FISH) or real-time C-reactive protein (RT-PCR) [11], although its intense turnover can hamper these detections.

TN tumours express other biomarkers such as p53, c-kit, claudin7, CK5/6, CK17, androgen receptors, PTEN, ALK and PDL-1 [12], demonstrating their heterogeneity [13]. The EGFR has a greater diversity of action, acting in the mesenchymal transformation of the epithelial cell [14], in addition to activating several signalling pathways with PI3K when dimerising with HER3, or with RAS when it binds to Grb2 proteins, or JAK when it binds to STAT proteins [15]. Among human epidermal receptors, only EGFR signals with RANK to up-regulate the AKT and ERK pathways [16]. EGFR is activated by EGF, which at a high concentration intensely internalises EGFR. In this condition, the cell adds ubiquitin molecules to EGFR [17]. This signalling can occur as a monomer or dimer with other ligands, such as amphiregulin present in extracellular vesicles, providing resistance to degradation [18]. The wild EGFR can signal through a non-canonical pathway [19], or even with chemokines such as CXCL8, but also may enable numerous negative regulations with the inhibition of EGFR [20] thus minimising the proliferative signalling [21].

Other proteins modulate the EGFR, either hindering internalisation, such as annexin A6 [22], or inducing endosomal recycling, such as the p53 protein when overexpressed, or the DRAM1 [23] and NSD2 proteins that exacerbate signalling [24], or with the Tinagl1 protein that suppresses several signalling pathways [25], modifying the action of anti-EGFR [9]. Oestrogen signalisation with EGFR results in the synthesis of the SDF-1 (CXCL12) and its receptor CXCR4, thus activating HER2, triggered by the family of Src kinases [26]. And Src kinases stimulate STAT3s which, again, intensify the modulation of EGFR [27], through intracellular signalling [28].

Overexpression of EGFR in tumour cells has been considered a poor prognostic factor in breast cancer for decades [29–31], with rare disagreements [32]. EGFR overexpression in breast tumours can be high, varying in intensity in different tumour subtypes [33, 34]. In TN neoplasms, EGFR overexpression in the tumour is found in 15%–30% of cases, with a worse prognosis especially in the presence of intense nuclear expression of EGFR [15].

The receptors of neoplastic cells can be measured in serum, through their soluble form as soluble HER-2 (sHER2), reflecting the expression of HER2 in the membrane [35]. The EGFR measured in tumour cells by IH or FISH [36] can also be measured in plasma or serum [37] and is routinely detected in patients with lung cancer [38]. The detection of EGFR as a protein expressed in the serum of breast cancer patients may be a non-invasive practice with better prognostic stratification.

This study aims to analyse the serological profile of soluble EGFR (sEGFR) in different breast cancer subgroups and its possible value as a prognostic marker, and to verify the relationship of sEGFR with clinicopathological variables and with the EGFR present in the tumour cell [tumour EGFR (tEGFR)].

## Methodology

A prospective study enrolling newly diagnosed breast cancer patients treated between 2015 and 2019 was conducted at the Oncology Sector of the Clinical Hospital of the Federal University of Uberlandia, Brazil, after approval by the Ethics Committee for Research on Humans (294,508/13). The healthy control group consisted of volunteers from this hospital. All participants read and signed the Informed Consent Form. This study did not interfere with the patients’ treatment.

The sample was categorised into four groups. Three groups of patients had a biopsy showing histologically infiltrating carcinoma of the breast, but had not yet undergone surgery or treatment with chemotherapy, radiotherapy and/or hormone therapy. Staging followed the* American Joint Committee on Cancer Seventh Edition* [39], grouping patients into early tumours (stages I/II), locally advanced tumours (stages III) and metastatic tumours (stages IV). The fourth group consisted of healthy volunteers, with BIRADS I and II mammograms performed within the last 12 months. A questionnaire was applied to obtain information on clinical aspects, family history, use of oral contraceptives and other drugs, hormone replacement therapies and smoking [40].

Exclusion criteria for patients were second neoplasm or another severe systemic disease, such as rheumatologic, cardiac, renal or hepatic, diagnosed at inclusion or during the study. For the control group, the exclusion criterion was the diagnosis of any neoplasm or serious systemic diseases mentioned above.

Peripheral blood samples were collected from the control group and, before any treatment of the patients, placed in collection tubes in separator gel and activator to accelerate coagulation. The sEGFR measurement was performed using the sandwich Enzyme-Linked Immunosorbent Assay (ELISA) kit #DY231 (R&D Systems) according to the manufacturer’s recommendations. The reading was performed in a GloMax Explorer (Promega). The sEGFR concentrations were determined by the equation obtained from the standard curve concentrations.

In addition, the paraffin blocks were submitted to histological sections of tumor lesions, stained with hematoxylin-eosin, selecting areas of greater neoplastic representation, and mounted for optimized analysis in tissue samples array (tissue microarray (TMA)) [41] of according to the routine of a high-demand pathology laboratory [42]. The antibody employed to detect tEGFR was Dako’s clone 31G7, analysed by two pathologists at different times. When the two disagreed in their analysis, a discussion was established until consensus was reached. In the absence of any reaction/staining, the result was negative. The presence of any reaction/staining was considered positive, ranging from 1% to 90%, stratified as follows: <10% weak positive; ≥10% and <40% intermediate positive; and ≥40% strong positive.

### Statistical analysis

Data were analysed using the software packages IBM SPSS 25.0, Jamovi 1.6.7 and GraphPad Prism 8.0. Statistical significance was defined as *p* < 0.05.

The Shapiro–Wilk statistical test verified data distribution, and descriptive data were represented according to the obtained distributions. Student’s *t*-test or Mann–Whitney U test was utilised to assess the difference between the two groups. ANOVA or Kruskal–Wallis was utilised to test the difference between more than two groups, with Tukey or Dunn’s *post hoc*, respectively.

To assess bivariate correlation in the presence of an ordinal or categorical variable, or in the presence of a continuous, non-symmetric/normal variable, Spearman’s correlation test was used. The association between interdependent categorical factors was evaluated using Pearson’s χ^2^ test. The association was considered positive (direct) when the adjusted standardised residuals had a value >(+2.0) and considered negative (indirect/inverse) when the value was <(–2.0).

The survival curve with continuous variables was used for determining the optimal cutoff points and prognostic factors of continuous variables. For survival analysis of categorical variables, the Kaplan–Meier estimator was employed to evaluate the proportionality of risks as a requirement for Cox regression.

## Results

This study included 206 patients with breast carcinoma and 89 healthy age-matched women. In a median follow-up period of 36.6 months, 47 deaths occurred. Twenty-six patients were not operated on, 20 for being metastatic and 6 due to early death, high surgical risk or progression during neoadjuvant chemotherapy. The results are reported according to the guidelines established by Strengthening the Reporting of Observational Studies in Epidemiology. Other clinical and therapeutic characteristics are shown in Table 1.

**Figure 1.**
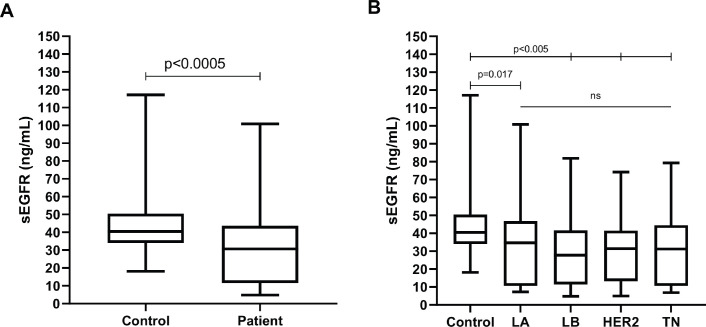
Boxplot representing the peripheral blood serum EGFR (sEGFR) levels (ng/mL) in (a): healthy volunteers (*n* = 89) versus breast cancer patients (*n* = 206) and (b): healthy volunteers versus breast cancer patients (*n* = 206), according to IH subtype (LA, Luminal A; LB, Luminal B; HER2, HER2/ERBB2 overpexression/amplification; TN, triple-negative).

The sEGFR level from patients (30.70 ng/mL, range: 4.78–100.85) differed significantly (Mann–Whitney U *p* < 0.0005) from healthy controls (40.45 ng/mL, range: 18.10–117.15) (Figure 1a). Significantly greater sEGFR levels were observed in healthy controls compared to all breast cancer subtypes (Kruskal–Wallis *p* < 0.0005; Dunn’s *p* at least < 0.05 in all pairwise comparisons), but no difference was observed among molecular subtypes (Dunn’s *p* > 0.999 for all pairwise comparisons) (Figure 1b).

Subsequently, survival analyses were performed to test whether sEGFR has any prognostic value. In accordance with the exploratory nature of this study, survival analyses for continuous variables were performed for the entire cohort as well as subgroup analyses to obtain the best sEGFR cutoff predictive for survival in each context.

**Figure 2.**
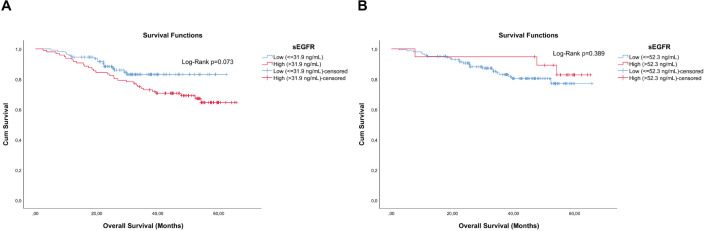
Cumulative survival curves by the KM estimator according to sEGFR levels. (a): Overall survival in the entire cohort (*n* = 206) and (b): non-TN breast cancer patients (*n* = 162). Each group was submitted to analysis for establishing the optimal sEGFR level cutoff with prognostic value. Lower sEGFR levels were marginally associated with increased overall survival (blue curve), compared to those with higher levels (red curve), but only in the entire cohort including TN breast cancer patients.

In the analysis of the sEGFR concentration among the 206 patients, although not significant (Log-Rank *p* = 0.073), a shorter overall survival (OS) was observed in those with the highest concentration of sEGFR (Figure 2a). Exclusion of the 44 TN patients resulted in a high increase of the *p*-value (Log-Rank *p* = 0.389) (Figure 2b).

The 96 patients with sEGFR > 31.9 ng/mL had a median OS of 30.27 months (range: 5.07–62.40), significantly lower (Mann–Whitney U *p* < 0.0005) than the 110 patients with sEGFR ≤ 31.9 ng/mL (median OS of 46.55 months (range: 2.47–65.63)). Again, when TN patients are excluded from these analyses, this difference in OS disappears (Mann–Whitney U *p* = 0.728). In the OS analysis, the sEGFR levels, alongside the immunohistochemistry type and stage, were associated with prognosis (Table 2). A higher risk of death was observed in patients with Luminal B tumours, and more intensely with TN tumours, compared to patients with lower risk such as Luminal A tumours, and also with patients with HER2^+^ tumours but using trastuzumab. The type of surgery did not interfere with OS (*p* = 0.324).

Analysing the expression of sEGFR in the IH subtypes, we found that in the subgroups of patients with Luminal A, Luminal B and HER2+ tumours, there was a non-significant (*p* > 0.05) difference in OS.

**Figure 3.**
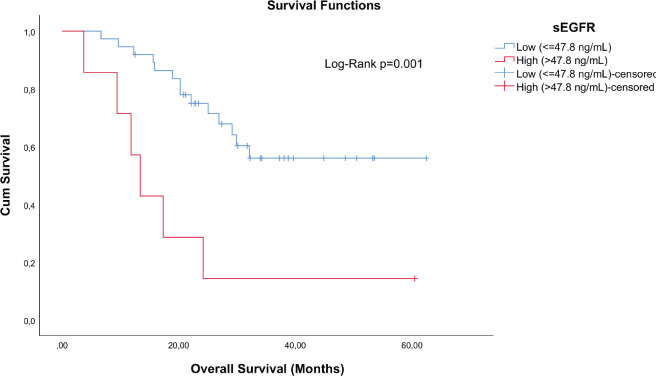
Cumulative survival curves by the KM estimator according to sEGFR levels in TN patients (*n* = 44). Lower sEGFR levels were associated with increased overall survival (blue curve), compared to those with higher levels (red curve).

TN breast cancer patients (*n* = 44) with sEGFR ≤ 47.8 ng/mL (37 patients) had a higher median OS (27.33 months (range: 6.63–62.40)) than those with sEGFR > 47.8 ng/mL (13.37 months (range: 3.67–60.43)) (Figure 3). By multivariable Cox regression, the high sEGFR (>47.8 ng/mL) experienced a death risk increase of more than 400% during the follow-up (adjusted HR: 5.149 (1.900–13.955), *p* = 0.001) (Table 3). The subgroup with low sEGFR expression, with longer survival between TN tumours, had lower OS than patients with Luminal A (*p* = 0.0004) and HER2^+^ (*p* = 0.0130) tumours, but with no OS difference in relation to patients with luminal B tumours (*p* = 0.0991).

Our analysis of patients with early tumours (I+II; *n* = 97) revealed that the TN subtype showed significantly reduced OS in univariable and multivariable analyses (Table 4). Patients with early tumours with sEGFR expression > 45.9 ng/mL presented, in univariable analysis, higher OS than those with lower sEGFR values (*p* = 0.045), but without significance in multivariable analysis (*p* = 0.051). And, again, when TN patients were excluded from these initial tumours, there was no significant difference in the analyses (univariable Cox regression *p* = 0.609). In patients in stages III (79 patients; univariable Cox regression *p* = 0.438) or IV (30 patients; univariable Cox regression *p* = 0.965), the OS was not influenced by high or low sEGFR expression.

There was no significant correlation of sEGFR with histological tumour grade (G1/G2/G3; Spearman’s rho of −0.003; *p* = 0.972), Ki67 (Spearman’s rho of −0.004; *p* = 0.956), BMI, tumour size (Spearman’s rho of −0.011; *p* = 0.876), lymph node metastasis (Spearman’s rho of −0.090; *p* = 0.225), nor stage (Spearman’s rho of −0.045; *p* = 0.522). Similarly, there was no significant difference in the median sEGFR levels in relation to the categories of histological tumour grade (Kruskal–Wallis *p* = 0.996), Ki67 (< or ≥14%) (Mann–Whitney U *p* = 0.518), tumour size (Kruskal–Wallis *p* = 0.246), lymph node metastasis (Kruskal–Wallis *p* = 0.372) nor stage (Kruskal–Wallis *p* = 0.822).

The initial tested cutoff (sEGFR > 31.9 ng/mL) was also not associated with histological grade (Pearson’s χ^2^: 0.129, *p* = 0.938), high ki67 (≥14%) levels (Pearson’s χ^2^: 0.567, *p* = 0.451), tumour size (Pearson’s χ^2^: 5.087, *p* = 0.166), lymph node metastasis (Pearson’s χ^2^: 4.734, *p* = 0.192), stage (Pearson’s χ^2^: 1.109, *p* = 0.775) nor immunohistochemistry subtype (Pearson’s χ^2^: 2.086, *p* = 0.555). Similar non-significant results were observed in the analysis of exclusive TN cancers by its obtained cutoff (>47.8 ng/mL).

The expression of tEGFR was analysed in 94 patients, equally representing all stages and IH subtypes, of which 66 specimens were negative and 28 (29.78%) were positive in ≥ 1% in tumour cells, with 11 weak positives, 6 intermediate positives and 11 strong positives. There was no correlation between tEGFR and sEGFR (Spearman’s rho of −0.022; *p* = 0.852). Negative or positive tEGFR had no impact on OS (Log-Rank *p* = 0.518). There was an inverse correlation between tEGFR and Oestrogen Receptor (ER) (Spearman’s rho of −0.752; *p* < 0.005). ER-negative breast cancers expressed more tEGFR (Mann–Whitney U *p* < 0.0001).

## Discussion

The IH subtypes and stages directly impact breast cancer prognosis. Compared to Luminal A and HER2^+^ (when properly treated with trastuzumab) subtypes, Luminal B and TN subtypes have shorter survivals. Also, locally advanced and metastatic tumours (stages III and IV) have significantly shorter OS than initial-stage ones [43–45]. In the current study, the survival of patients was similar to other treatment centres, varying by prognostic characteristics and geographic regions [46]. Patients with more aggressive HER2+ tumours receiving anti-HER2 therapy had more prolonged survival than those with Luminal B tumours. On the other hand, predictive factors and an effective target therapy remain lacking in patients with TN breast tumours, who continue to face poorer survival.

Only 94 paraffin blocks of tumours were analysed, but with representation proportional to the stages and IH subtypes. The technique for analysing tEGFR by TMA is reproducible like the individualised IH [47]. Our tEGFR analysis was hampered by several technical issues. We planned to analyse the expression of EGFR in the tumour in at least 50% of the cases under study, already foreseeing the difficulty of accessing this material since many patients undergo biopsy or undergo surgery in other services and then are referred to our centre for treatment. Of the 116 paraffin blocks accessed in 22 samples, there was no viable tissue, resulting from fine needle biopsies or damaged by non-ideal storage [48]. The expression of tEGFR did not interfere with OS, perhaps because of our small sample number. A similar study [49] but with a larger number of patients showed that tEGFR overexpressed in TN tumors, detected by immunohistochemistry [30], also worsened survival. We also did not specify the location of tEGFR, as the nuclear expression has a worse prognosis than tEGFR expression in the cytoplasmic membrane [50]. We demonstrated a significant inverse correlation between tEGFR and ER expression, although without specifying whether the tumour cells were ERβ or ERα, which have different expressions of tEGFR [51]. Despite these deficiencies, we also did not find a correlation between tEGFR and sEGFR, similar to other studies [52, 53]. Due to the difficulties presented – such as scarcity of material, difficulty in ideal storage and the possibility of constant recycling of tEGFR – the measurement of sEGFR by the ELISA method may allow the analysis of this tumour aggressiveness parameter, with greater precision.

The sEGFR concentration in all patients was significantly lower than in healthy women, a result that is conflicting in the literature and lacks a rational explanation [54–56]. But perhaps in healthy women, there is a necessity for positive regulations for growth and physiological division, without the numerous possible negative regulations, such as the internalisation of these overexpressed receptors and other EGFR inhibitions in an attempt to minimise the proliferative signalling of the neoplasia. Therefore, sEGFR is not a biomarker for population screening. The sEGFR measurements in cancer patients are also contradictory but can be explained by the methodological disparities among the studies.

Analyses of women with metastatic breast cancer have reported low levels of sEGFR, but with normal sHER2, being related to shorter survival [57]; or high concentrations associated with longer survival [54]; or absence in the survival correlation with the sEGFR [52, 53]. In early breast tumours, there is a study correlating low sEGFR expression with lower survival [58]. We did not find this difference in the survival of patients with early or advanced tumours.

We suggest that sEGFR is not a promising biomarker to stratify higher or lower risk patients among those with luminal A, B or HER2+ tumours. However, in the subgroup with TN tumours, the sEGFR distinguished in early and advanced tumours, with statistical significance, two risk groups. In the low expression of sEGFR, there was longer survival, but significantly lower than the OS of Luminal A or HER2+ patients and equal to the survival of patients with Luminal B tumours. This TN group with low sEGFR expression would be the group with the least poor prognosis. And a second TN group with an even worse prognosis is those patients with high sEGFR expression and a four-fold greater death risk. In EGFR gene signalling, there is overexpression of other proteins, and when there is simultaneous signalling of the HER2 gene, the Snail [59] protein is overexpressed, with an evident deterioration in survival. There are improvements in this prognosis by pharmacological modulation, such as the efficient use of anti-HER2 therapy, as probably occurred in the patients in the current study who expressed EGFR and HER2. We did not measure the sHER2 protein, but together with the sEGFR, perhaps the treatment response can be measured in patients with metastatic breast cancer [60].

Despite progress in the treatment of women with TN breast tumours, there is a need to stratify among TN tumours those with better or worse prognosis, de-escalating treatment in women with less aggressive tumours. We need both greater individualisation and more predictive markers for the proper treatment of TN tumours. Perhaps the use of PARP inhibitors in mutated BRCA1/2 should be consolidated in the adjuvant setting [61], or when the ideal association is found for taxane and anthracycline such as carboplatin, pembrolizumab or atezolizumab in neoadjuvant treatment [62]. And in the metastatic setting, the benefit of immunotherapy associated with chemotherapy has already been demonstrated [63, 64] with promising new formulations such as sacituzumab govitecan-hziy [65]. And research continues targeting EGFR in TN tumours. The use of cetuximab was disappointing, due to its toxicity [66] and few objective responses [67]. But the combination of cetuximab with panitumumab proved to be effective in EGFR degradation and tumour reduction in an experimental study [68]. And new presentations of cetuximab, in *in vitro* studies, linked to nanoparticles minimised treatment resistance [69], through promising associations with bioconjugated molecules [70, 71], or with titanium, which hinders the action of ligands on EGFR [72].

Another possible inhibition of EGFR is through the use of TKIs. Preclinical studies have shown that gefitinib increased the response to carboplatin and docetaxel [73]; doxazosin reduced EGFR expression [74]; epertinib inhibited EGFR and HER2 [75]; almonertinib inhibited the multi-resistance of cancer cells [76]. The association of erlotinib and metformin minimised signalling [77] and the inhibition of EGFR and PI3K increased the sensitivity to irradiation [78]. Everolimus associated with gefitinib inhibited the PI3K/AKT/mTOR pathways [79], and gefitinib with anastrozole showed an objective [80] but irregular response to TKI due to the heterogeneity of EGFR [81]. A small clinical study in patients with advanced bowel cancer, having sEGFR measured, used FOLFOX6 and gefitinib, demonstrating a more objective response in those with higher sEGFR [82]. Research studies directed to the management of EGFR [83], including new gene therapies [84], demonstrated the feasibility of the suppression of the Notch3 gene with reduced internalisation of EGFR [85], or the construction of mutant forms of the EGF ligand blocking this receptor [86], or the genetic silencing by small RNAs of interference (siRNA) against EGFR [87]. New therapies, new antibody formations or new associations with TKIs signal the importance of adequately measuring EGFR, both as a prognostic biomarker, and as a treatment predictor, in women with TN breast tumours.

This study has several limitations. Despite its prospective nature and assessment of patients from the year 2015 onwards, most of whom received adequate treatments according to current guidelines, the short follow-up period and low sample size make the external validity very low, thus requiring caution when extrapolating these data to any and all patients with breast cancer, especially the TN subtype. Therefore, the results must be understood as exploratory, requiring multicentre data and the follow-up of a greater number of patients for a longer period of time.

## Conclusion

EGFR measurement is inexpensive and straightforward and can be measured in TN breast cancer. Increased sEGFR expression significantly increased the death risk in TN tumours, but not in Luminals or HER2+. Future studies should confirm our findings and allow different therapeutic options depending on the serum concentration of this biomarker, reconfiguring two large groups of TN tumours: one with a less-bad prognosis and another TN group with an even worse prognosis.

## Conflicts of interest

The authors declare that there are no conflicts of interest.

## Funding statement

This work was supported by Grupo Luta Pela Vida. The authors received no financial support for authorship and/or publication of this article.

## Data

Data is deposited in Mendeley Data (DOI: 10.17632/xxt6mc2pk7.3)

## Figures and Tables

**Table 1.** Clinical, laboratory and treatment characteristics of patients (*n* = 206).

Factor	*n* (%)	Mean/median
Overall survival	206 (100)	36.65 months (2.47–65.63)
Age	206 (100)	55.17 years (±12.37)
Age		
<70 years	180 (87.4)	
≥70 years	26 (12.6)	
Deaths		
No	159 (77.2)	
Yes	47 (22.8)	
Stage		
I	18 (8.7)	
II	79 (38.3)	
III	79 (38.3)	
IV	30 (14.6)	
Molecular subtype		
Luminal A	44 (21.4)	
Luminal B	78 (37.9)	
HER2	40 (19.4)	
TN	44 (21.4)	
Hormone receptor expression		
No	61 (29.6)	
Yes	145 (70.4)	
Histological grade		
G1	16 (7.8)	
G2	123 (59.7)	
G3	64 (31.1)	
NR	3 (1.5)	
Surgery		
No	26 (12.6)	
Yes	180 (87.4)	
Surgical approach		
Conservative	100/180 (55.6)	
Mastectomy/Radical	80/180 (43.4)	
Surgical margin		
Negative	160/180 (88.9)	
Positive	19/180 (10.6)	
NR	1/180 (0.5)	
Chemotherapy		
No	32 (15.5)	
Neoadjuvant	107 (52.0)	
Adjuvant	67 (32.5)	
Radiation therapy		
No	39 (18.9)	
Yes	167 (81.1)	
Hormonal therapy		
No	8/145 HR^+^ (5.5)	
Yes	137/145 HR^+^ (94.5)	
Trastuzumab (HER2^+^)		
No	4 (10.0)	
Yes	36 (90.0)	

HR: hazard ratio; NR: not reported

**Table 2.** Univariable and multivariable analyses of overall survival of breast cancer patients (*n* = 206).

Factor	Univariable		Multivariable	
	HR (95%CI)	*p*	HR (95%CI)	*p*
sEGFR (ng/mL)				
≤31.9	1		1	
>31.9	1.741 (0.943–0.216)	0.076	2.976 (1.577–5.616)	0.001
Age				
<70 years	1			
≥70 years	1.197 (0.535–2.674)	0.662		
Stage				
I/II	1		1	
III	3.098 (1.408–6.817)	0.005	3.371 (1.502–7.563)	0.003
IV	3.176 (0.852–11.841)	0.085	11.674 (5.062–26.924)	0.0005
Histological grade				
G1/2	1			
G3	2.207 (1.244–3.916)	0.007		
Molecular subtype				
Luminal A	1		1	
Luminal B	2.646 (1.691–94.566)	0.013	12.428 (1.651–93.546)	0.014
HER2	8.069 (0.993–65.598)	0.051	4.841 (0.590–39.700)	0.142
TN	31.473 (4.208–235.415)	0.001	40.741 (5.332–11.274)	0.0005

**Table 3.** Multivariable analysis of overall survival of patients with TN breast tumour (*n* = 44).

Factor	Univariable		Multivariable	
	HR (95%CI)	*p*	HR (95%CI)	*p*
sEGFR (ng/mL**)**				
≤47.8	1		1	
>47.8	4.584 (1.732–12.133)	0.002	5.149 (1.900–13.955)	0.001
Stage				
I/II	1		1	
III/IV	2.358 (0.906–6.278)	0.078	2.670 (0.996–7.159)	0.051

**Table 4.** Univariable and multivariable analyses of overall survival in subgroups of patients with early (stages I + II) breast tumours (*n* = 97).

Factor	Univariable		Multivariable	
	HR (95%CI)	*p*	HR (95%CI)	*p*
EGFR (ng/mL) ≤45.9	1		1	
>45.9	3.873 (1.028–14.586)	0.045	3.743 (0.996–14.074)	0.051
Age <70 years	1			
≥70 years	1.041 (0.130–8.340)	0.970		
T T1/2	1			
T3	3.176 (0.852–11.841)	0.085		
N N0	1			
N1	0.396 (0.050–3.166)	0.382		
Histological grade G1/2	1			
G3	5.248 (1.408 – 19.564)	0.014		
Molecular subtype Non-TN	1		1	
TN	14.401 (2.898–71.561)	0.001	10.427 (2.569–42.319)	0.001
